# The genome sequence of the Cloaked Minor, *Mesoligia furuncula* (Denis & Schiffermüller, 1775)

**DOI:** 10.12688/wellcomeopenres.19537.1

**Published:** 2023-06-16

**Authors:** Douglas Boyes, Gavin R. Broad, Peter W.H. Holland, University of Oxford and Wytham Woods Genome Acquisition Lab, Natural History Museum Genome Acquisition Lab, Darwin Tree of Life Barcoding collective, Wellcome Sanger Institute Tree of Life programme, Wellcome Sanger Institute Scientific Operations: DNA Pipelines collective, Tree of Life Core Informatics collective, Darwin Tree of Life Consortium

**Affiliations:** 1 UK Centre for Ecology & Hydrology, Wallingford, England, UK; 2 Natural History Museum, London, England, UK; 3 University of Oxford, Oxford, England, UK

**Keywords:** Mesoligia furuncula, Cloaked Minor, genome sequence, chromosomal, Lepidoptera

## Abstract

We present a genome assembly from an individual female *Mesoligia furuncula* (the Cloaked Minor; Arthropoda; Insecta; Lepidoptera; Noctuidae). The genome sequence is 889.6 megabases in span. Most of the assembly is scaffolded into 31 chromosomal pseudomolecules, including the Z sex chromosome. The mitochondrial genome has also been assembled and is 15.3 kilobases in length. Gene annotation of this assembly on Ensembl identified 21,903 protein coding genes.

## Species taxonomy

Eukaryota; Metazoa; Ecdysozoa; Arthropoda; Hexapoda; Insecta; Pterygota; Neoptera; Endopterygota; Lepidoptera; Glossata; Ditrysia; Noctuoidea; Noctuidae; Xyleninae; *Mesoligia*; *Mesoligia furunculi* (Denis & Schiffermüller, 1775) (NCBI:txid997551).

## Background

The Cloaked Minor *Mesoligia furuncula* is a small moth in the family Noctuidae (wingspan 22–28 mm) found widely across central and northern Europe, with scattered records from Russia, Kyrgyzstan, Uzbekistan, Kazakhstan, China and Japan ( GBIF Secretariat, 2022). The moth is common across southern England, northern France, Belgium and the Netherlands, where it is found in grassland, scrub, open woodland and suburban areas ( De Prins & Steeman, 2022; Randle *et al.*, 2019). In Scotland, Northern Ireland, Wales and Ireland the species is more coastal in its distribution, being primarily associated with dunes and coastal grassland ( GBIF Secretariat, 2022).

The forewing colouration is variable, but a consistent feature is a division of the ground colour into two near equal regions: a darker basal region (the ‘cloak’) and a paler cream, buff or brown distal region. Colour variants were originally considered different species before conspecificity was recognized ( Newman, 1869). The adult moth is on the wing in July and August, with the larvae feeding on grasses including sheep’s-fescue *Festuca ovina*, tufted hair-grass *Deschampsia cespitosa* and false oat-grass *Arrhenatherum elatius* ( De Prins & Steeman, 2022; Waring *et al.*, 2017). There is a single generation per year in northern Europe; the larva overwinters before recommencing feeding in spring and pupating in a chamber at the base of the food plant.

The genome of *M. furuncula* was sequenced as part of the Darwin Tree of Life project. The assembled sequence will be useful in understanding adaptations to grass feeding and the genetic basis of colour polymorphism, and contribute to comparative genomic studies across Lepidoptera.

## Genome sequence report

**Figure 1.**
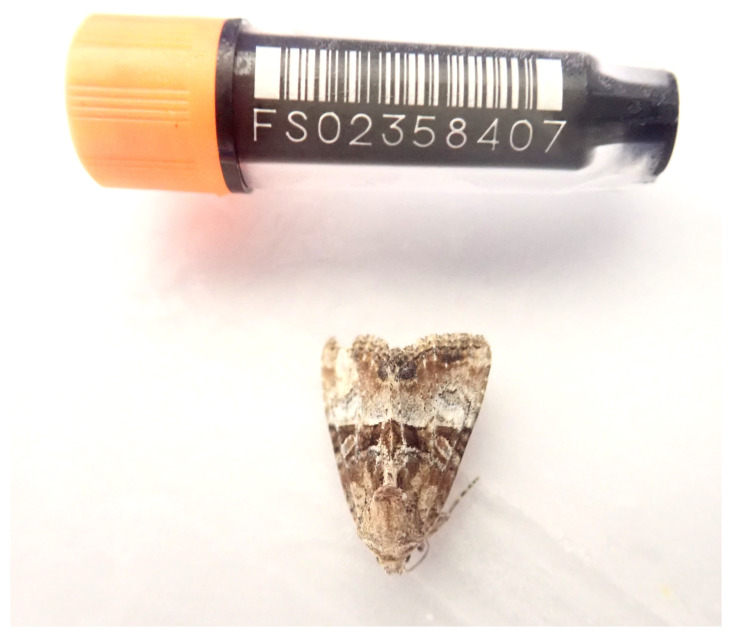
Photograph of the *Mesoligia furuncula* (ilMesFuru1) specimen used for genome sequencing.

The genome was sequenced from one female *Mesoligia furuncula* ( Figure 1) collected from Wytham Woods, Oxfordshire, UK (latitude 51.77, longitude –1.33). A total of 24-fold coverage in Pacific Biosciences single-molecule HiFi long reads and 62-fold coverage in 10X Genomics read clouds were generated. Primary assembly contigs were scaffolded with chromosome conformation Hi-C data. Manual assembly curation corrected 284 missing joins or mis-joins and removed 27 haplotypic duplications, reducing the assembly length by 1.44% and the scaffold number by 67.25%, and increasing the scaffold N50 by 62.83%.

**Figure 2.**
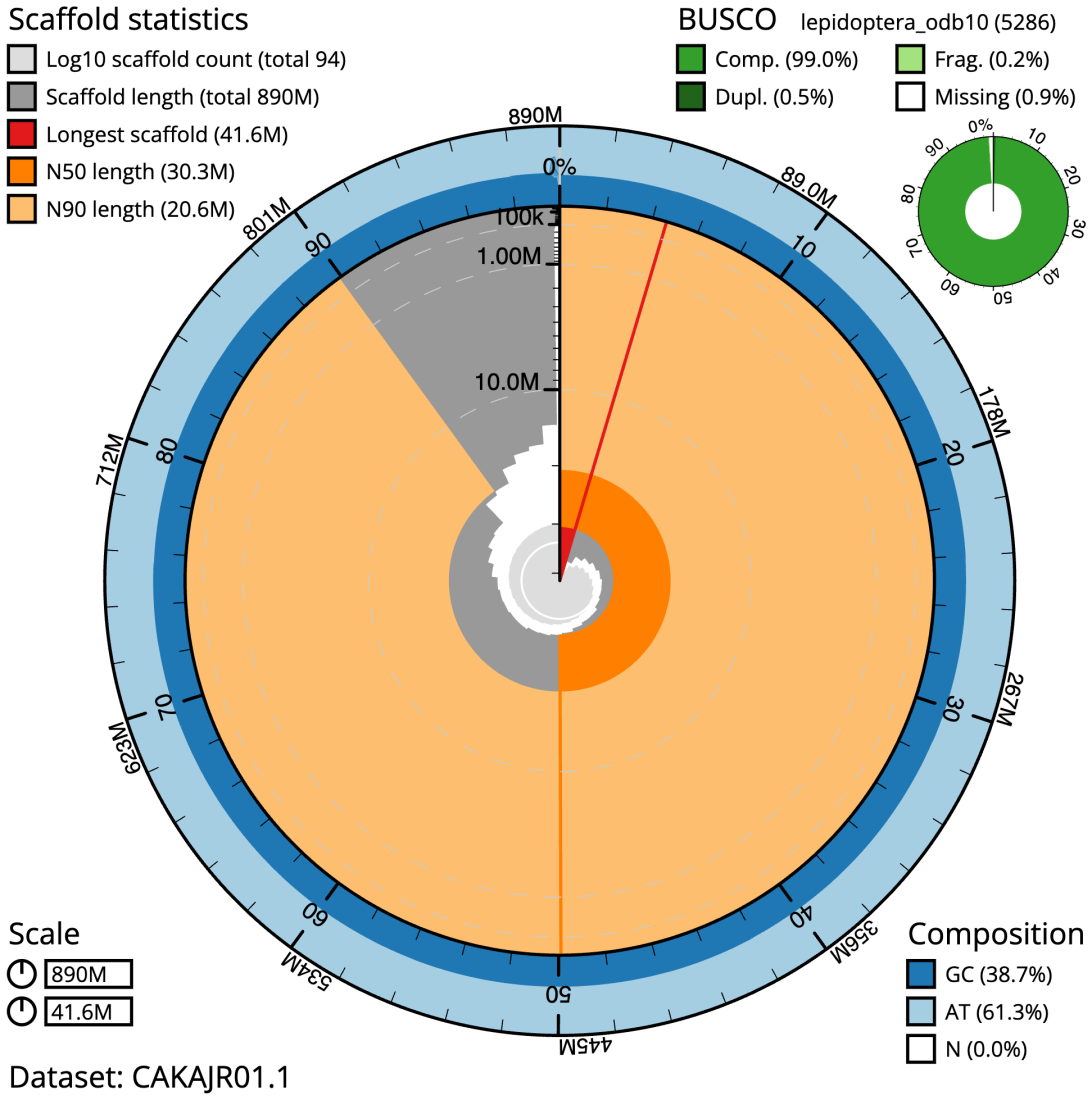
Genome assembly of *Mesoligia furuncula*, ilMesFuru1.1: metrics. The BlobToolKit Snailplot shows N50 metrics and BUSCO gene completeness. The main plot is divided into 1,000 size-ordered bins around the circumference with each bin representing 0.1% of the 889,627,388 bp assembly. The distribution of scaffold lengths is shown in dark grey with the plot radius scaled to the longest scaffold present in the assembly (41,648,309 bp, shown in red). Orange and pale-orange arcs show the N50 and N90 scaffold lengths (30,263,258 and 20,641,088 bp), respectively. The pale grey spiral shows the cumulative scaffold count on a log scale with white scale lines showing successive orders of magnitude. The blue and pale-blue area around the outside of the plot shows the distribution of GC, AT and N percentages in the same bins as the inner plot. A summary of complete, fragmented, duplicated and missing BUSCO genes in the lepidoptera_odb10 set is shown in the top right. An interactive version of this figure is available at https://blobtoolkit.genomehubs.org/view/ilMesFuru1.1/dataset/CAKAJR01.1/snail.

The final assembly has a total length of 889.6 Mb in 93 sequence scaffolds with a scaffold N50 of 30.3 Mb ( Table 1). Most (99.64%) of the assembly sequence was assigned to 31 chromosomal-level scaffolds, representing 30 autosomes and the Z sex chromosome. Chromosome-scale scaffolds confirmed by the Hi-C data are named in order of size ( Figure 2– Figure 5; Table 2). The sex was designated as female due to half coverage of the Z chromosome. Linkage has been observed in the Hi-C map between chromosomes 8 and 25. This linkage is specific, such that an alternative karyotype could be constructed {8A:8B,25A:25B} and {8B:25A,8A,25B}. No support for this fusion between chromosome 8 and 25 can be seen in the PacBio reads which derive from a different sample to the Hi-C.

**Table 1.** Genome data for *Mesoligia furuncula*, ilMesFuru1.1.

Project accession data		
Assembly identifier	ilMesFuru1.1	
Species	*Mesoligia furuncula*	
Specimen	ilMesFuru1	
NCBI taxonomy ID	997551	
BioProject	PRJEB46328	
BioSample ID	SAMEA7701289	
Isolate information	ilMesFuru1, female, whole organism (genome sequencing) ilMesFuru2, head (Hi-C scaffolding) ilMesFuru3, whole organism (RNA sequencing)	
Assembly metrics *		*Benchmark*
Consensus quality (QV)	60.2	≥ *50*
*k*-mer completeness	100%	≥ *95%*
BUSCO **	C:99.0%[S:98.5%,D:0.5%], F:0.2%,M:0.9%,n:5,286	C * ≥ 95%*
Percentage of assembly mapped to chromosomes	99.64%	≥ *95%*
Sex chromosomes	Z chromosome	*localised homologous pairs*
Organelles	Mitochondrial genome assembled	*complete single alleles*
Raw data accessions		
PacificBiosciences SEQUEL II	ERR6808010, ERR6907889, ERR6939247	
10X Genomics Illumina	ERR6688571–ERR6688574	
Hi-C Illumina	ERR6688570	
PolyA RNA-Seq Illumina	ERR9435012	
Genome assembly		
Assembly accession	GCA_916614155.1	
*Accession of alternate haplotype*	GCA_916611985.1	
Span (Mb)	889.6	
Number of contigs	423	
Contig N50 length (Mb)	4.7	
Number of scaffolds	93	
Scaffold N50 length (Mb)	30.3	
Longest scaffold (Mb)	41.7	
Genome annotation		
Number of protein-coding genes	21,903	
Number of gene transcripts	22,079	

* Assembly metric benchmarks are adapted from column VGP-2020 of “Table 1: Proposed standards and metrics for defining genome assembly quality” from ( Rhie *et al.*, 2021). ** BUSCO scores based on the lepidoptera_odb10 BUSCO set using v5.3.2. C = complete [S = single copy, D = duplicated], F = fragmented, M = missing, n = number of orthologues in comparison. A full set of BUSCO scores is available at https://blobtoolkit.genomehubs.org/view/ilMesFuru1.1/dataset/CAKAJR01.1/busco.

**Figure 3.**
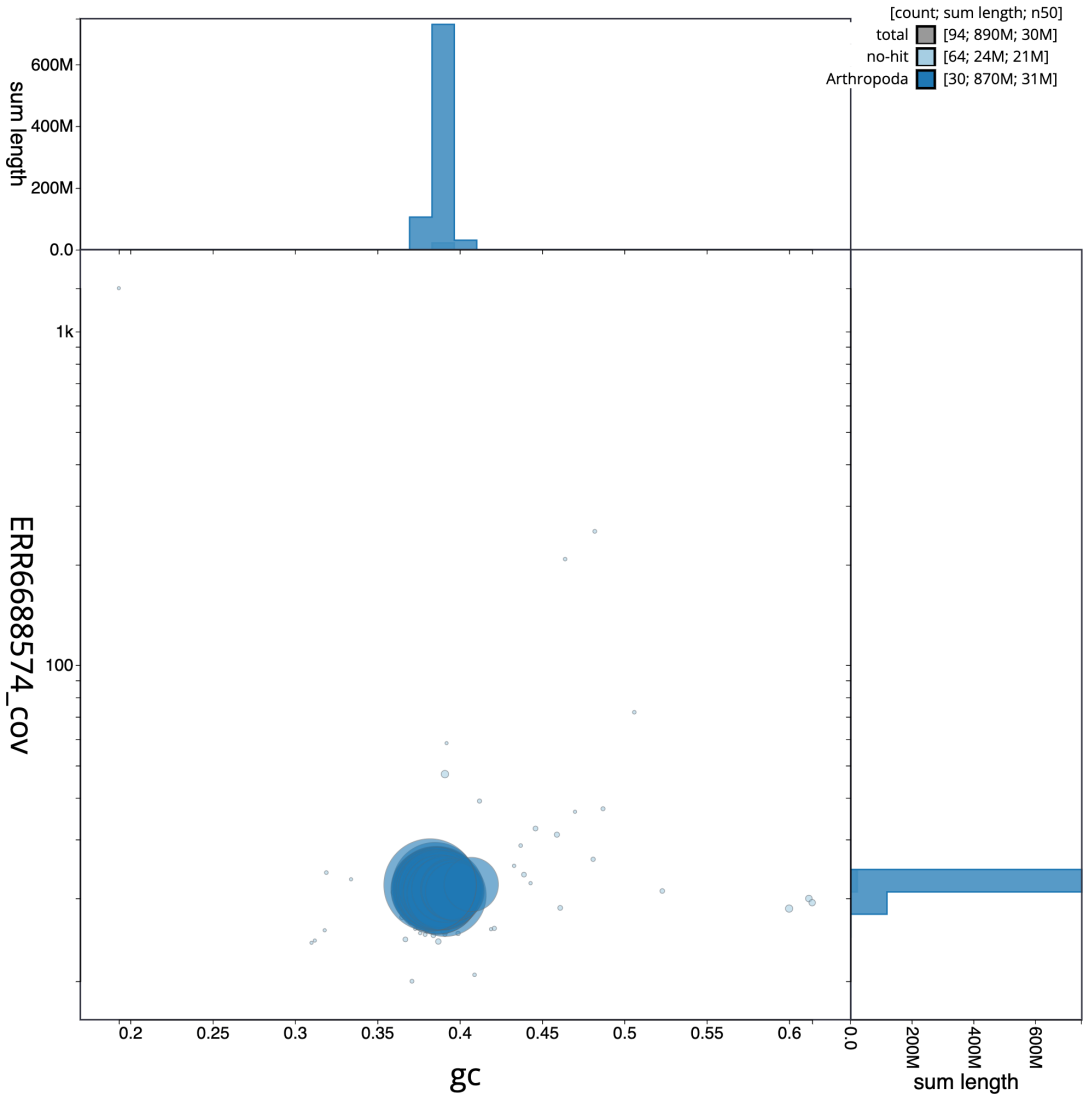
Genome assembly of *Mesoligia furuncula*, ilMesFuru1.1: BlobToolKit GC-coverage plot. Scaffolds are coloured by phylum. Circles are sized in proportion to scaffold length. Histograms show the distribution of scaffold length sum along each axis. An interactive version of this figure is available at https://blobtoolkit.genomehubs.org/view/ilMesFuru1.1/dataset/CAKAJR01.1/blob.

**Figure 4.**
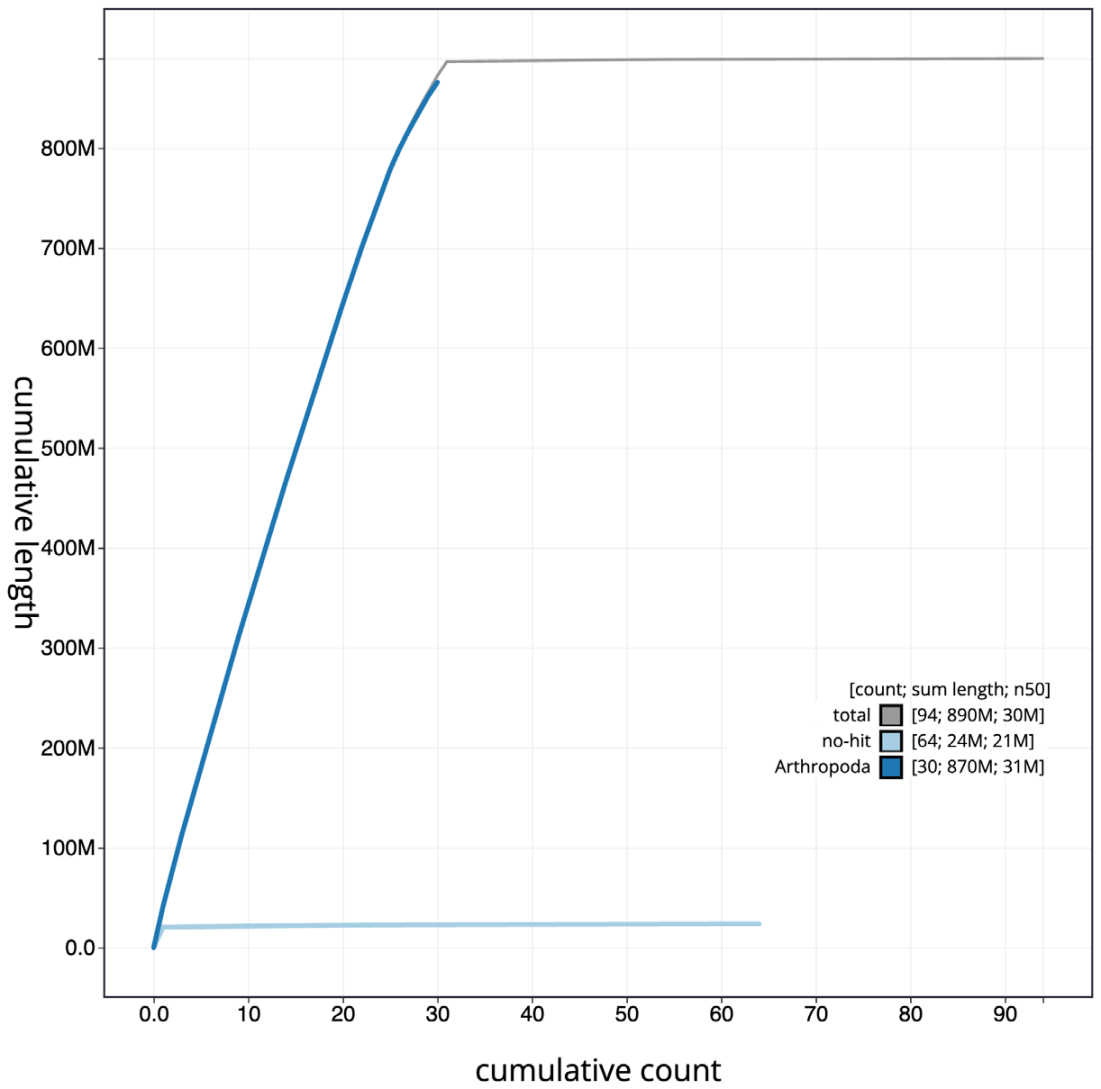
Genome assembly of *Mesoligia furuncula*, ilMesFuru1.1: BlobToolKit cumulative sequence plot. The grey line shows cumulative length for all scaffolds. Coloured lines show cumulative lengths of scaffolds assigned to each phylum using the buscogenes taxrule. An interactive version of this figure is available at https://blobtoolkit.genomehubs.org/view/ilMesFuru1.1/dataset/CAKAJR01.1/cumulative.

**Figure 5.**
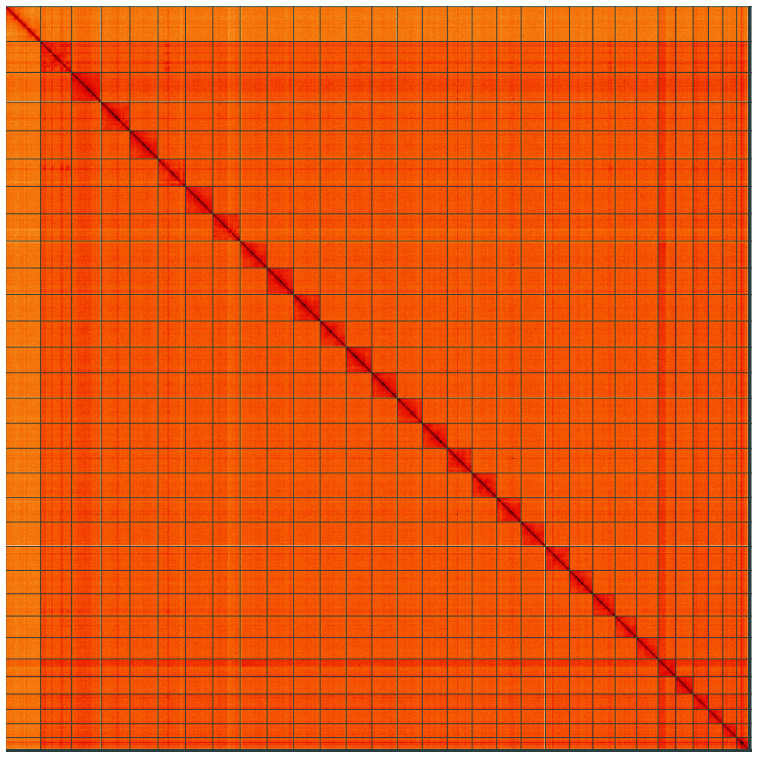
Genome assembly of *Mesoligia furuncula*, ilMesFuru1.1: Hi-C contact map of the ilMesFuru1.1 assembly, visualised using HiGlass. Chromosomes are shown in order of size from left to right and top to bottom. An interactive version of this figure may be viewed at https://genome-note-higlass.tol.sanger.ac.uk/l/?d=HtJsaWOeRfirYpmZg7BeWQ.

**Table 2.** Chromosomal pseudomolecules in the genome assembly of *Mesoligia furuncula*, ilMesFuru1.

INSDC accession	Chromosome	Size (Mb)	GC%
OU744791.1	1	36.99	38.6
OU744792.1	2	35.24	38.5
OU744793.1	3	34.29	38.7
OU744794.1	4	33.51	38.5
OU744795.1	5	32.93	39.1
OU744796.1	6	32.6	38.7
OU744797.1	7	32.51	38.7
OU744798.1	8	32.24	38.3
OU744799.1	9	31.65	38.4
OU744800.1	10	31.58	38.3
OU744801.1	11	31.18	38.7
OU744802.1	12	30.6	38.5
OU744803.1	13	30.26	38.6
OU744804.1	14	30.02	38.6
OU744805.1	15	29.86	38.4
OU744806.1	16	29.45	38.5
OU744807.1	17	29.42	38.5
OU744808.1	18	29.2	38.6
OU744809.1	19	29.06	38.8
OU744810.1	20	28.76	39.1
OU744811.1	21	27.69	38.6
OU744812.1	22	26.63	38.8
OU744813.1	23	25.75	38.8
OU744814.1	24	25.41	39
OU744815.1	25	21.26	39.1
OU744816.1	26	20.64	39
OU744817.1	27	18.35	39.5
OU744818.1	28	17.12	39.5
OU744819.1	29	16.55	39.7
OU744820.1	30	14.05	40.7
OU744790.1	Z	41.65	38.2
OU744821.1	MT	0.02	19.5
-	unplaced	3.12	43

While not fully phased, the assembly deposited is of one haplotype. Contigs corresponding to the second haplotype have also been deposited. The mitochondrial genome was also assembled and can be found as a contig within the multifasta file of the genome submission.

The estimated Quality Value (QV) of the final assembly is 60.2 with *k*-mer completeness of 100%, and the assembly has a BUSCO v5.3.2 completeness of 99.0% (single = 98.5%, duplicated = 0.5%), using the lepidoptera_odb10 reference set ( *n* = 5,286).

Metadata for specimens, spectral estimates, sequencing runs, contaminants and pre-curation assembly statistics can be found at https://links.tol.sanger.ac.uk/species/997551.

## Genome annotation report

The *M. furuncula* genome assembly GCA_916614155.1 was annotated using the Ensembl rapid annotation pipeline ( Table 1; https://rapid.ensembl.org/Mesoligia_furuncula_GCA_916614155.1/Info/Index). The resulting annotation includes 22,079 transcribed mRNAs from 21,903 protein-coding genes.

## Methods

### Sample acquisition and nucleic acid extraction

Two adult *M. furuncula* were collected from Wytham Woods, Oxfordshire (biological vice-county: Berkshire), UK (latitude 51.77, longitude –1.33) by Douglas Boyes (University of Oxford) on 25 June 2020. Individual ilMesFuru1 (specimen Ox000519) was used for acquisition of the genome sequence; individual ilMesFuru3 (specimen Ox000520) was used for RNA sequencing. An adult *M. furuncula* was collected from Hever Castle, Kent, UK (latitude 51.88, longitude 0.12) by Gavin Broad (Natural History Museum, London) on 7 August 2020; this individual (ilMesFuru2, specimen NHMUK010635087) was used for scaffolding using Hi-C.

DNA was extracted at the Tree of Life laboratory, Wellcome Sanger Institute (WSI). The ilMesFuru1 sample was weighed and dissected on dry ice. Whole organism tissue was disrupted using a Nippi Powermasher fitted with a BioMasher pestle. High molecular weight (HMW) DNA was extracted using the Qiagen MagAttract HMW DNA extraction kit. Low molecular weight DNA was removed from a 20 ng aliquot of extracted DNA using the 0.8X AMpure XP purification kit prior to 10X Chromium sequencing; a minimum of 50 ng DNA was submitted for 10X sequencing. HMW DNA was sheared into an average fragment size of 12–20 kb in a Megaruptor 3 system with speed setting 30. Sheared DNA was purified by solid-phase reversible immobilisation using AMPure PB beads with a 1.8X ratio of beads to sample to remove the shorter fragments and concentrate the DNA sample. The concentration of the sheared and purified DNA was assessed using a Nanodrop spectrophotometer and Qubit Fluorometer and Qubit dsDNA High Sensitivity Assay kit. Fragment size distribution was evaluated by running the sample on the FemtoPulse system.

RNA was extracted from whole organism tissue of ilMesFuru3 in the Tree of Life Laboratory at the WSI using TRIzol, according to the manufacturer’s instructions. RNA was then eluted in 50 μl RNAse-free water and its concentration assessed using a Nanodrop spectrophotometer and Qubit Fluorometer using the Qubit RNA Broad-Range (BR) Assay kit. Analysis of the integrity of the RNA was done using Agilent RNA 6000 Pico Kit and Eukaryotic Total RNA assay.

### Sequencing

Pacific Biosciences HiFi circular consensus and 10X Genomics read cloud DNA sequencing libraries were constructed according to the manufacturers’ instructions. Poly(A) RNA-Seq libraries were constructed using the NEB Ultra II RNA Library Prep kit. DNA and RNA sequencing was performed by the Scientific Operations core at the WSI on Pacific Biosciences SEQUEL II (HiFi), Illumina HiSeq 4000 (RNA-Seq) and Illumina NovaSeq 6000 (10X) instruments. Hi-C data were also generated from head tissue of ilMesFuru2, using the Arima2 kit and sequenced on the Illumina NovaSeq 6000 instrument.

### Genome assembly, curation and evaluation

Assembly was carried out with Hifiasm ( Cheng *et al.*, 2021) and haplotypic duplication was identified and removed with purge_dups ( Guan *et al.*, 2020). One round of polishing was performed by aligning 10X Genomics read data to the assembly with Long Ranger ALIGN, calling variants with FreeBayes ( Garrison & Marth, 2012). The assembly was then scaffolded with Hi-C data ( Rao *et al.*, 2014) using SALSA2 ( Ghurye *et al.*, 2019). The assembly was checked for contamination as described previously ( Howe *et al.*, 2021). Manual curation was performed using HiGlass ( Kerpedjiev *et al.*, 2018) and Pretext ( Harry, 2022). The mitochondrial genome was assembled using MitoHiFi ( Uliano-Silva *et al.*, 2022), which runs MitoFinder ( Allio *et al.*, 2020) or MITOS ( Bernt *et al.*, 2013) and uses these annotations to select the final mitochondrial contig and to ensure the general quality of the sequence. To evaluate the assembly, MerquryFK was used to estimate consensus quality (QV) scores and *k*-mer completeness ( Rhie *et al.*, 2020). The genome was analysed within the BlobToolKit environment ( Challis *et al.*, 2020) and BUSCO scores ( Manni *et al.*, 2021; Simão *et al.*, 2015) were calculated. Table 3 contains a list of software tool versions and sources.

**Table 3.** Software tools: versions and sources.

Software tool	Version	Source
BlobToolKit	4.0.7	https://github.com/blobtoolkit/ blobtoolkit
BUSCO	5.3.2	https://gitlab.com/ezlab/busco
FreeBayes	1.3.1-17- gaa2ace8	https://github.com/freebayes/ freebayes
Hifiasm	0.15.3	https://github.com/chhylp123/hifiasm
HiGlass	1.11.6	https://github.com/higlass/higlass
Long Ranger ALIGN	2.2.2	https://support.10xgenomics.com/ genome-exome/software/pipelines/ latest/advanced/other-pipelines
Merqury	MerquryFK	https://github.com/thegenemyers/ MERQURY.FK
MitoHiFi	2	https://github.com/marcelauliano/ MitoHiFi
PretextView	0.2	https://github.com/wtsi-hpag/ PretextView
purge_dups	1.2.3	https://github.com/dfguan/purge_ dups
SALSA	2.2	https://github.com/salsa-rs/salsa

### Genome annotation

The BRAKER2 pipeline ( Brůna *et al.*, 2021) was used in the default protein mode to generate annotation for the *Mesoligia furuncula* assembly (GCA_916614155.1) in Ensembl Rapid Release.

### Ethics and compliance issues

The materials that have contributed to this genome note have been supplied by a Darwin Tree of Life Partner. The submission of materials by a Darwin Tree of Life Partner is subject to the Darwin Tree of Life Project Sampling Code of Practice. By agreeing with and signing up to the Sampling Code of Practice, the Darwin Tree of Life Partner agrees they will meet the legal and ethical requirements and standards set out within this document in respect of all samples acquired for, and supplied to, the Darwin Tree of Life Project. All efforts are undertaken to minimise the suffering of animals used for sequencing. Each transfer of samples is further undertaken according to a Research Collaboration Agreement or Material Transfer Agreement entered into by the Darwin Tree of Life Partner, Genome Research Limited (operating as the Wellcome Sanger Institute), and in some circumstances other Darwin Tree of Life collaborators.

## Data Availability

European Nucleotide Archive: *Mesoligia furuncula* (cloaked minor). Accession number PRJEB46328; https://identifiers.org/ena.embl/PRJEB46328. ( Wellcome Sanger Institute, 2021) The genome sequence is released openly for reuse. The *Mesoligia furuncula* genome sequencing initiative is part of the Darwin Tree of Life (DToL) project. All raw sequence data and the assembly have been deposited in INSDC databases. Raw data and assembly accession identifiers are reported in Table 1.

## Author information

Members of the University of Oxford and Wytham Woods Genome Acquisition Lab are listed here: https://doi.org/10.5281/zenodo.4789928.

Members of the Natural History Museum Genome Acquisition Lab are listed here: https://doi.org/10.5281/zenodo.4790043.

Members of the Darwin Tree of Life Barcoding collective are listed here: https://doi.org/10.5281/zenodo.4893703.

Members of the Wellcome Sanger Institute Tree of Life programme are listed here: https://doi.org/10.5281/zenodo.4783585.

Members of Wellcome Sanger Institute Scientific Operations: DNA Pipelines collective are listed here: https://doi.org/10.5281/zenodo.4790455.

Members of the Tree of Life Core Informatics collective are listed here: https://doi.org/10.5281/zenodo.5013541.

Members of the Darwin Tree of Life Consortium are listed here: https://doi.org/10.5281/zenodo.4783558.
