# Supplementary material for: The genome sequence of the Cloaked Minor, Mesoligia furuncula (Denis & Schiffermüller, 1775)
Source: Wellcome Open Res. 2023 Jun 16;8:251. [Version 1] doi: 10.12688/wellcomeopenres.19537.1 (PMC11162523; doi:10.12688/wellcomeopenres.19537.1)
Supplement: Peer review history

# Reviewer report: Reviewer response for version 1

**DOI:** 10.21956/wellcomeopenres.21641.r83735

**Published:** 2024-06-08

**Authors:** Frederik Hendrickx

**Affiliations:** 1 Royal Belgian Institute of Natural Sciences, Brussels, Belgium

The paper clearly describes the quality and completeness of the genome, which is also of very high quality. I have two suggestions for improvement:

 1. Within the abstract, I suggest to replace 'Most of the assembly is scaffolded..." directly by the exact percentage i.e. "99.64% of the assembly is scaffolded..."

 2. Within the part "Genome sequence report", there is one paragraph that is less clear and concerns the linkage between chromosomes 8 and 25. First, it is unclear what is meant with "This linkage is specific..." and it would be helpful to elaborate a bit more what is exactly meant with "specific". Second, the HiC contact map (Fig. 5) does not show this linkage. Third, the authors describe that PacBio reads do not support this linkage and attribute this to the fact that another individual was used. However, I wonder how reasonable it is that the PacBio assembly would reveal this linkage? In sum, some more explanation about this linkage and what it potentially entails would be welcome.

 3. I suggest to add the chromosome numbers in Fig. 5 (also helpful to interpret the previous point).

Are sufficient details of methods and materials provided to allow replication by others?

Yes

Is the rationale for creating the dataset(s) clearly described?

Yes

Are the datasets clearly presented in a useable and accessible format?

Yes

Are the protocols appropriate and is the work technically sound?

Yes

Reviewer Expertise:

Evolutionary genomics

I confirm that I have read this submission and believe that I have an appropriate level of expertise to confirm that it is of an acceptable scientific standard.

# Reviewer report: Reviewer response for version 1

**DOI:** 10.21956/wellcomeopenres.21641.r70104

**Published:** 2023-12-28

**Authors:** Kuppusamy Sivasankaran

**Affiliations:** 1 Entomology Research Institute, Loyola College, Chennai, Tamil Nadu, India

I appreciate the authors for putting together the entire genome sequence of *Mesoligia furuncula* (Denis & Schiffermuller, 1775). The authors sequenced about 889 mega bases in the genome assembly using 93 scaffolds. The sequence has been correctly submitted by the authors to the public database. The phylogenomic researchers will find value in this robust data.  

 The first sentence of the abstract can be changed as “The genome of an individual female *Mesoligia furuncula *(Insect: Lepidoptera: Noctuidae) was sequenced and assembled”

 There is a spelling error in the species name in the “species taxonomy”. *furunculi* was typed instead of *furuncula*. Correct the spelling of the species name.

 Under the subheading “Genome sequence report” first paragraph seventh line” with chromosome conformation” It can be changed as “with chromosome confirmation”

 The manuscript is well prepared, and it can be accepted for indexing.

Are sufficient details of methods and materials provided to allow replication by others?

Yes

Is the rationale for creating the dataset(s) clearly described?

Yes

Are the datasets clearly presented in a useable and accessible format?

Yes

Are the protocols appropriate and is the work technically sound?

Yes

Reviewer Expertise:

Analyzing the phylogenetic relationship of the lepidopteran Noctuoid moths using mitochondrial genome sequence

I confirm that I have read this submission and believe that I have an appropriate level of expertise to confirm that it is of an acceptable scientific standard.
